# Reaction-Multi Diffusion Model for Nutrient Release and Autocatalytic Degradation of PLA-Coated Controlled-Release Fertilizer

**DOI:** 10.3390/polym9030111

**Published:** 2017-03-22

**Authors:** Sayed Ameenuddin Irfan, Radzuan Razali, KuZilati KuShaari, Nurlidia Mansor

**Affiliations:** 1 Department of Fundamental and Applied Sciences, Universiti Teknologi PETRONAS, 32610 Seri Iskandar, Perak Darul Ridzuan, Malaysia; 2 Department of Chemical Engineering, Universiti Teknologi PETRONAS, 32610 Seri Iskandar, Perak Darul Ridzuan, Malaysia; kuzilati_kushaari@utp.edu.my (K.K.); nurlidia_mansor@utp.edu.my (N.M.)

**Keywords:** controlled-release fertilizers, numerical Laplace inversion method, reaction-diffusion, poly(lactic acid)

## Abstract

A mathematical model for the reaction-diffusion equation is developed to describe the nutrient release profiles and degradation of poly(lactic acid) (PLA)-coated controlled-release fertilizer. A multi-diffusion model that consists of coupled partial differential equations is used to study the diffusion and chemical reaction (autocatalytic degradation) simultaneously. The model is solved using an analytical-numerical method. Firstly, the model equation is transformed using the Laplace transformation as the Laplace transform cannot be inverted analytically. Numerical inversion of the Laplace transform is used by employing the Zakian method. The solution is useful in predicting the nutrient release profiles at various diffusivity, concentration of extraction medium, and reaction rates. It also helps in explaining the transformation of autocatalytic concentration in the coating material for various reaction rates, times of reaction, and reaction-multi diffusion. The solution is also applicable to the other biodegradable polymer-coated controlled-release fertilizers.

## 1. Introduction

Controlled-release fertilizer (CRF) contains many important sources of nutrients, such as nitrogen, phosphorus, and potassium, that are crucial to plants [1,2]. The CRF granules are coated with polymers [3], organic polymers, biopolymers, natural biomolecule materials, and nanocomposites [2]. The coating materials play an important role in nutrient release profiles by providing the obstacles to the mixing of nutrients with water and help in the water retention of the granules [4]. Biodegradable materials have shown good results in maintaining the optimal control release rates and are environmentally-friendly, as well [5]. Some of the biodegradable material [6], such as starch [6], polyvinyl alcohol [7], 3,5-pyridinedicarboxylic acid *N*-oxide [8], and poly(lactic acid) [9] and nanocomposites and nanomaterials [10,11] are used as a coating of CRF granules. PLA, a natural synthetic polymer available in abundance, has shown good results in biodegradability, controlled-release rates, and is used extensively in drug delivery and implants [12,13,14,15]. 

Biodegradation of biopolymers is a complicated process as it occurs through scission of the main chains and side chains of macromolecules. Biodegradation is induced by many factors, such as thermal activation, hydrolysis, biological activity or enzymes, oxidation, and radiolysis [16]. PLA is an aliphatic polyester and is prone to chemical hydrolysis of ester backbones in the aqueous environment. Hydrolysis of PLA is autocatalytic in nature because the degradation starts when PLA comes in contact with water molecules naturally [17]. For the case of PLA, the hydrolytic degradation reaction is heterogeneous because it degrades faster on the surface than the core [17].

The chemical reaction for the hydrolytic degradation of PLA is given by Equation (1) [18]. The hydrolytic degradation of PLA has been studied by Grizzi et al. [19] in order to understand the effect of size distribution on the degradation. Later, Speranza et al. [18]. studied the effect of thermal and hydrolytic degradation of PLA. They also calculated the reaction rate constants for thermal and hydrolytic degradation. The kinetics of hydrolytic degradation of PLA is given by [20] the detailed of chemical kinetics related to hydrolytic degradation as mentioned in the present study. It was found that up to 95% of PLA is hydrolyzed to form water soluble lactic acid.

(1) $$H_{2}O+\mathrm{ester}↔\mathrm{COOH}+-\mathrm{OH}$$

This makes it difficult to design and optimize the nutrient release applications. There have been many studies with regard to PLA modeling, but most of the papers dealt with the drug delivery applications in pharmaceuticals [15]. There is limited research that deals with the modeling of PLA-coated CRF. For the case of CRF, the nutrient release rate from granules with different size and diffusivity depends mainly on release regime. Hence, the study of nutrient release should necessarily be undertaken in conjunction with the knowledge of polymer degradation. 

Modeling of CRF in the single diffusion mechanism was considered by Jarrel et al. [21,22,23]. They discovered that the nutrient release profile was coming from the sulfur-coated urea. Later, Al-Zahrani took into account the single solute diffusion mechanism to predict the nutrient release from CRF [24]. Shaviv et al. [25,26] considered non-Fickian diffusion to model the nutrient release from CRF. In these studies, the nutrient release was divided into three stages; that is, lag period, linear release, and extended release stages for singular granule and for the population of the granules. Lu et al. [27] developed an explicit mathematical analysis of release from coated particle using multi-diffusion phenomenon. Trinh et al. [28,29,30,31] used the multi-diffusion model by employing the finite element method to investigate the nutrient release in the decay period (extended release stage). They also studied the effect of imperfect coating on nutrient release in both soil and water.

For the case of modeling of autocatalytic degradation reaction of PLA in the literature, the models used the homogeneous degradation before the starting of the erosion [32,33]. A PLA-based reaction-diffusion model has been proposed by Wang et al. [34] for autocatalytic degradation for polymer implants in the form of plates and cylinders. The model can be used to other geometries by solving numerically using finite element analysis for the degradation map. The other models of polymer degradation [17,35,36] assumed that hydrolytic degradation follows the pseudo-first-order kinetics to include the autocatalytic effect. 

To the best of our knowledge with regard to CRF modeling, there is no model available in the literature, which can predict the nutrient release profile for the reaction-multi diffusion system. The purpose of the current study is to investigate the effect of reaction rates coupled with multi-diffusion on the nutrient release profiles and to obtain insight of autocatalytic degradation of the polymer coating. For this purpose, an analytical-numerical solution was derived from a reaction-multi diffusion model by employing the Laplace and Zakian method. This model can also be extended to other studies of CRF granules using biodegradable polymers as a coating material. 

## 2. Mathematical Model

**Figure 1:**
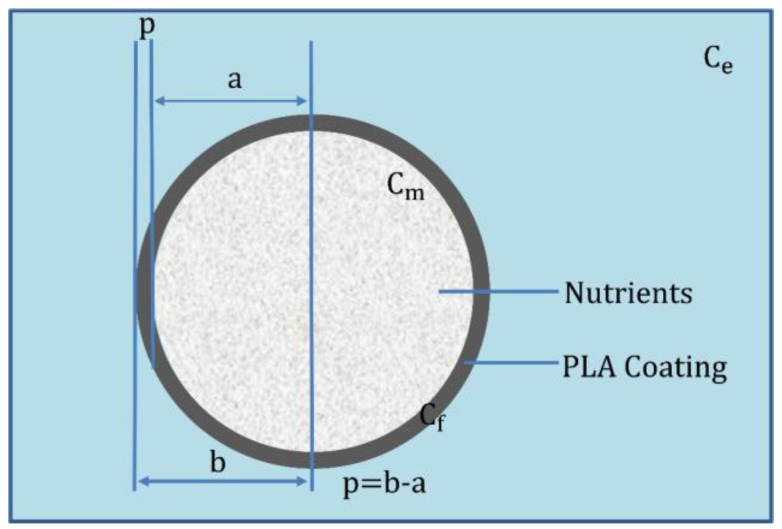
Schematic of the CRF.

A spherical, coated fertilizer granule has been considered as portrayed in the schematic diagram (Figure 1). It has a radius (*a*), uniform coating thicknesses (*p*), and a radius of the coated granule (*b*). The initial nutrient concentration in the granule (*C*_0_) which is, in turn, less than nutrient saturation concentration (*C_s_*). The nutrient concentration inside the granule, coating film, and extraction medium are represented by *C_m_*(*r*, *t*), *C_f_*(*r*, *t*), and *C_e_*, respectively. The diffusion coefficient of the granule and coating thickness are denoted as *D_m_* and *D_f_*, respectively.

When the PLA-coated granules are subjected to dissolution in wet soil, the water molecules penetrate through the coating membrane, and dissolve the nutrient which gives rise to the osmotic pressure inside the coating shell. The nutrient then diffuses from the dissolved core, through the coating membrane, and into the external environment. This chemical phenomenon is represented by the mathematical equations in Equations (2) and (3). 

The basic equations of multi-diffusion model along with reaction in a sphere are given as follows [37]:

(2) $$\frac{\partial \left(rC_{m}\right)}{\partial t}=D_{m}\frac{\partial^{2}\left(rC_{m}\right)}{\partial r^{2}},\;0\leq r<a$$

(3) $$\frac{\partial \left(rC_{f}\right)}{\partial t}=D_{f}\frac{\partial^{2}\left(rC_{f}\right)}{\partial r^{2}}+R_{v},\;a<r<b$$

The initial conditions are [27]:

(4a) $$C_{m}\left(r,0\right)=C_{0},\;C_{0}<C_{s}$$

(4b) $$C_{f}\left(r,0\right)=0,$$

(4c) $$C_{e}\left(0\right)=0$$

and boundary conditions are [27]:

(5a) $$C_{m}\left(0,t\right)\;\mathrm{is}\;\mathrm{finite}$$

(5b) $$K_{a}C_{f}\left(a,t\right)=C_{m}\left(a,t\right),$$

(5c) $$D_{m}{\left(\frac{\partial C_{m}}{\partial r}\right)}_{r=a}=D_{f}{\left(\frac{\partial C_{f}}{\partial r}\right)}_{r=b},$$

(5d) $$K_{b}C_{f}\left(b,t\right)=C_{e}\left(t\right),$$

(5e) $$4\pi b^{2}D_{f}{\left(\frac{\partial C_{f}}{\partial r}\right)}_{r=b}=V_{e}\left(\frac{\partial C_{e}}{\partial t}\right).$$

$K_{a}$ and $K_{b}$ are the constant values used in the partition. $V_{e}$ is the volume of extraction medium in which the nutrient dissolves. In Equation (3), $R_{v}$ is given as the net generation of species per volume. The chemical reaction for interest is autocatalysis of PLA degradation. For the PLA autocatalysis, when the concentration of the ester bonds and water is constant, it follows the pseudo-first order rate law [38]. Siparsky et al. [17] conducted an experimental study on PLA hydrolysis in aqueous medium and concluded that the PLA hydrolytic degradation shows good agreement with pseudo-first-order chemical kinetics. However, once the reaction is taken into consideration, the concentrations vary depending upon time and reaction rate. Hence:

(6) $$R_{v}=k\left(rC_{f}\right)$$

For the purpose of generality, the above problem is solved using the non-dimensional technique as defined in Equations (7a)–(7c):

(7a) $$\theta_{m}≅\frac{C_{m}}{C_{0}},\;\theta_{f}≅\frac{C_{f}}{C_{0}},\;and\;\theta_{e}≅\frac{C_{e}}{C_{0}}$$

(7b) $$\tau ≅\frac{tD_{m}}{a^{2}},\;\eta ≅\frac{r}{a}$$

(7c) $$D_{r}≅\frac{D_{f}}{D_{m}},\;V_{r}≅\frac{V_{e}}{\frac{4\pi b^{3}}{3}},\;and\;l≅\frac{b}{a}$$

The conservation equation is given by Equations (8) and (9):

(8) $$\frac{\partial \left(\eta \theta_{m}\right)}{\partial \tau }=\frac{\partial^{2}\left(\eta \theta_{m}\right)}{\partial \eta^{2}}$$

(9) $$\frac{\partial \left(\eta \theta_{f}\right)}{\partial \tau }=D_{r}\frac{\partial^{2}\left(\eta \theta_{f}\right)}{\partial \eta^{2}}+k\left(\eta \theta_{f}\right)$$

The initial and boundary conditions are also transformed as represented in Equations (10a)–(10h):

(10a) $$\theta_{m}=1,$$

(10b) $$\theta_{f}=0,$$

(10c) $$\theta_{e}=0,$$

and

(10d) $$\theta_{m}\left(0,\tau \right)\;\mathrm{is}\;\mathrm{finite},$$

(10e) $$K_{a}\theta_{f}\left(1,\tau \right)=\theta_{m}\left(1,\tau \right),$$

(10f) $${\left(\frac{\partial \theta_{m}}{\partial \eta }\right)}_{\eta =1}=D_{r}{\left(\frac{\partial \theta_{f}}{\partial \eta }\right)}_{\eta =1},$$

(10g) $$K_{b}\theta_{f}\left(l,\tau \right)=\theta_{e}\left(\tau \right),$$

(10h) $${\left(\frac{\partial \theta_{f}}{\partial \eta }\right)}_{\eta =l}=-\frac{V_{r}l}{3D_{r}}\left(\frac{\partial \theta_{e}}{\partial \tau }\right).$$

### 2.1. Analytical Solution of the Model

To solve the above set of equations represented in Equations (8) and (9) with the help of initial and boundary conditions given from Equations (10a)–(10h). The proposed solution is given by using the Laplace transform, which is defined as:

(11) $$\bar{\theta_{i}}\left(\eta ,s\right)=\overset{\infty }{\underset{0}{\int }}\theta_{i}\left(\eta ,\tau \right)e^{-s\tau }d\tau ,\;i=m,f,e$$

By using the above definition of Laplace transform, Equations (8) and (9) can be transformed as:

(12) $$\frac{d^{2}\left(\eta {\bar{\theta }}_{m}\right)}{d\eta^{2}}=s\left(\eta {\bar{\theta }}_{m}\right)-\eta ,$$

(13) $$\frac{d^{2}\left(\eta {\bar{\theta }}_{f}\right)}{d\eta^{2}}=\frac{s}{D_{r}}\left(\eta {\bar{\theta }}_{f}\right)-\frac{k}{sD_{r}}\left(\eta {\bar{\theta }}_{f}\right),$$

Boundary conditions are also transformed and written as:

(14a) $${\bar{\theta }}_{m}\left(0,s\right)\;\mathrm{is}\;\mathrm{finite},$$

(14b) $$K_{a}{\bar{\theta }}_{f}\left(1,s\right)={\bar{\theta }}_{m}\left(1,s\right),$$

(14c) $${\left(\frac{\partial {\bar{\theta }}_{m}}{\partial \eta }\right)}_{\eta =1}=D_{r}{\left(\frac{\partial {\bar{\theta }}_{f}}{\partial \eta }\right)}_{\eta =1},$$

(14d) $$K_{b}{\bar{\theta }}_{f}\left(l,s\right)={\bar{\theta }}_{e}\left(s\right),$$

(14e) $${\left(\frac{\partial {\bar{\theta }}_{f}}{\partial \eta }\right)}_{\eta =l}=-\frac{V_{r}l}{3D_{r}}s{\bar{\theta }}_{e}\left(s\right).$$

The solution of Equations (12) and (13) is given by the following results. The detailed derivation of the solution is not shown here due to lengthy steps. In the current paper, only significant steps are displayed. Thus, Equations (12) and (13) can be transformed into Equations (15) and (16), as shown below:

(15) $${\bar{\theta }}_{m}=\frac{1}{s}+\frac{A_{1}\sin h\sqrt{s}\eta +B_{1}\cos h\sqrt{s}\eta }{\eta },$$

(16) $${\bar{\theta }}_{f}=\frac{A_{2}\sin h\sqrt{\frac{-k+s^{2}}{sD_{r}}}\eta +B_{2}\cos h\sqrt{\frac{-k+s^{2}}{sD_{r}}}\eta }{\eta }.$$

where $A_{1},\;A_{2},\;B_{1},\;\mathrm{and}\;B_{2}$ are the constants to be determined using the initial and boundary conditions from Equations (14a)–(14e). By using Equation (14a) in Equation (15), we get $B_{1}=0$. Then, Equation (15) can be reduced to Equation (17):

(17) $${\bar{\theta }}_{m}=\frac{1}{s}+\frac{A_{1}\sin h\sqrt{s}\eta }{\eta }.$$

To find the constants *A*_1_, *A*_2_, and *B*_2_, the detailed procedure is presented from Equations (A1)–(A7) as presented in the Appendix A. 

The final set of equations after substituting the constants are given by Equations (18)–(20):

(18) $${\bar{\theta }}_{m}=\frac{1}{s}+\frac{D_{r}}{\eta s\left(g_{1}\left(s\right)g_{4}\left(s\right)-g_{2}\left(s\right)g_{3}\left(s\right)\right)}[\left[\left(l-1\right)\sqrt{\frac{-k+s^{2}}{sD_{r}}}+\frac{V_{r}K_{b}l^{2}}{3}\left(\frac{-k+s^{2}}{sD_{r}}\right)\sqrt{\frac{-k+s^{2}}{sD_{r}}}\right]\cos h\sqrt{\frac{-k+s^{2}}{sD_{r}}}\left(l-1\right)+l\left(\frac{-k+s^{2}}{sD_{r}}\right)-1+\frac{V_{r}K_{b}l^{2}}{3}\left(\frac{-k+s^{2}}{sD_{r}}\right)\sin h\sqrt{\frac{-k+s^{2}}{sD_{r}}}\left(l-1\right)]$$

(19) $${\bar{\theta }}_{f}=\frac{\left(\sqrt{s}\cos h\sqrt{s}-\sin h\sqrt{s}\right)}{\eta s\left(g_{1}\left(s\right)g_{4}\left(s\right)-g_{2}\left(s\right)g_{3}\left(s\right)\right)}[\left[1-\frac{V_{r}K_{b}l^{2}}{3}\left(\frac{-k+s^{2}}{sD_{r}}\right)\right]\sin h\sqrt{\frac{-k+s^{2}}{sD_{r}}}\left(l-\eta \right)\;-l\sqrt{\frac{-k+s^{2}}{sD_{r}}}\cos h\sqrt{\frac{-k+s^{2}}{sD_{r}}}\left(l-\eta \right)]$$

(20) $${\bar{\theta }}_{e}=-K_{b}\sqrt{\frac{-k+s^{2}}{sD_{r}}}\frac{\left(\sqrt{s}\cos h\sqrt{s}-\sin h\sqrt{s}\right)}{s\left(g_{1}\left(s\right)g_{4}\left(s\right)-g_{2}\left(s\right)g_{3}\left(s\right)\right)}.$$

### 2.2. Numerical Inversion of Laplace Transform

The above equations have to be inversed to obtain the solution. However, as these equations are complex and do not have a pole at *s* = 0, it is very difficult to obtain the inverse Laplace using the residue theorem [39]. The above problems can be solved by using numerical inversion of the Laplace transformation given by Zakian [40]. Zakian has developed an explicit formula for numerical inversion using a weighted function to approximate the time domain function.

(21) $$\bar{f}\left(t\right)=\frac{2}{t}\overset{N}{\underset{j=1}{\sum }}Re\left\{K_{j}F\left(\frac{\alpha_{j}}{t}\right)\right\}$$

where $K_{j}$ and $\alpha_{j}$ are the constant in complex conjugate pairs. *N* is the number of terms used in the summation. $\bar{f}\left(t\right)$ approaches f(t) only when *N* reaches ∞. The previous study has shown that the truncated error is negligible when *N* = 5 [41]. The constant values for $K_{j}\;$and $\alpha_{j}$ are shown in Table 1.

The dimensionless cumulative release of *g*(*τ*) is equal to $M_{t}/M_{\infty }$, which is equal to $\theta_{e}\left(\tau \right)/\theta_{e}\left(\infty \right)$. At infinity, the reaction rate of autocatalysis degradation is negligible. Hence, $\theta_{e}\left(\infty \right)$ is calculated as given by Lu et al. [27], which can be represented in Equation (22). The autocatalytic concentration profiles are obtained by solving the Equation (19):

(22) $$\theta_{e}\left(\infty \right)=\frac{1}{\frac{K_{a}}{K_{b}}+(l^{3}-1)/K_{b}+V_{r}l^{3}}$$

## 3. Results and Discussion

### 3.1. Nutrient Release Profiles

**Figure 2:**
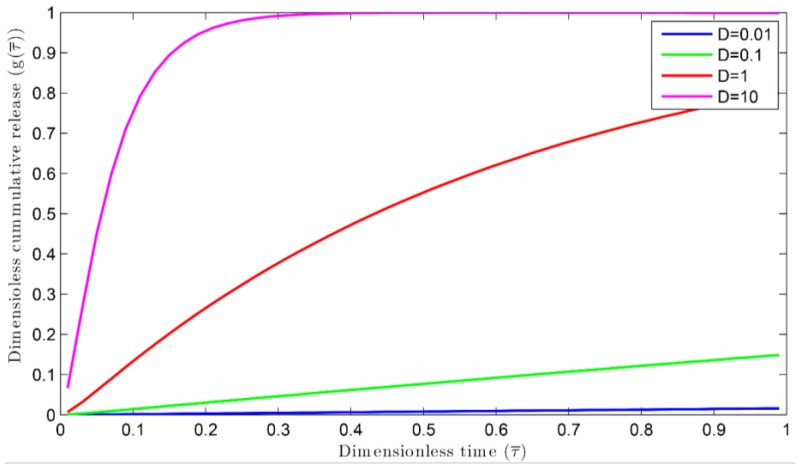
Dimensionless cumulative release versus dimensionless time for different diffusion coefficients.

**Figure 3:**
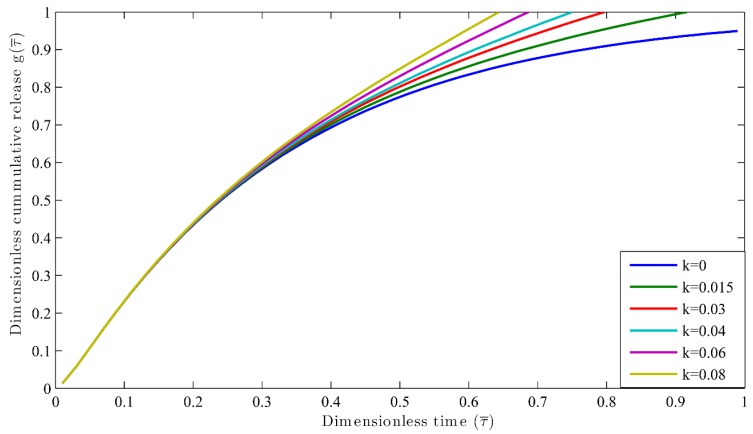
Dimensionless cumulative release versus dimensionless time for different reaction constants at $V_{r}=1$.

**Figure 4:**
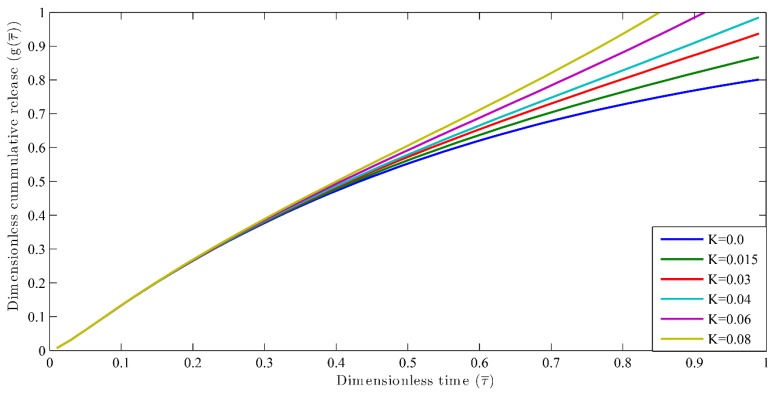
Dimensionless cumulative release versus dimensionless time for different reaction constants at $V_{r}=10$.

The simulation of reaction-multi diffusion has been carried out for a dimensionless coating thickness l=1, different volume of extraction medium, $V_{r}=1,\;3,\;10,\;K_{a}=K_{b}=1$, and different dimensionless diffusivity, $D_{r}=0.01,\;0.1,\;1,\;\mathrm{and}\;10$. The nutrient release profiles have been presented from Figure 2, Figure 3 and Figure 4. In Figure 2, the release profiles have been portrayed only for multi-diffusion. In this case, the reaction rate constant (*k*) has been considered as zero. The model equations are presented by Equations (23a) and (23b). These equations can be solved with appropriate initial and boundary conditions from Equations (10a)–(10h). The diffusivity of a material can hold the nutrient from release. To see these effects, various $D_{r}$ are chosen, whereby $D_{r}=0.01,\;0.1,\;1,\;\mathrm{and}\;10$, while $V_{r}=10$. The dimensionless nutrient release with dimensionless time has been shown in Figure 2. As the diffusivity increases, the nutrient release rate also increases [43]. The time taken for 99% of nutrient release ($\tau_{99}$) at $D_{r}=10$ is less than $\tau_{99}$ when $D_{r}=1$. The same profile can be seen when $D_{r}=0.1$ and 0.01.

(23a) $$\frac{\partial \left(\eta \theta_{m}\right)}{\partial \tau }=\frac{\partial^{2}\left(\eta \theta_{m}\right)}{\partial \eta^{2}}$$

(23b) $$\frac{\partial \left(\eta \theta_{f}\right)}{\partial \tau }=D_{r}\frac{\partial^{2}\left(\eta \theta_{f}\right)}{\partial \eta^{2}}$$

Nutrient release profiles by considering both reaction and multi-diffusion systems have been obtained for different reaction rates, that is, *k* = 0, 0.015, 0.03, 0.02, 0.04, 0.06, 0.08 day^−1^. The simulation was carried out for constant diffusivity, whereby$\;D_{r}=1$, l=1, and $V_{r}=10$. The results are shown in the Figure 3. For the case of $D_{r}=0.1$ and $V_{r}=1,$ the results are shown in Figure 4. The value of the extraction medium plays an important role. For example, when the value of diffusivity is less than 1 [27], its result can be clearly seen from Figure 3 and Figure 4. It can also be inferred that when the reaction rate *k* = 0, the $\tau_{90}$ is larger for both the cases as explained in Figure 3 and Figure 4 as compared to $\tau_{90}$ when *k* = 0.015. When the reaction rate constant (*k*) increases, $\tau_{90}$ decreases. This indicates more release of nutrients in the shorter duration of time. When the initial stage of nutrient release is taken as 30%, the time taken $\left(\tau_{30}\right)$ is the same for all the possible k values. These phenomena occur because the release of nutrient at the initial stage is taking place when the degradation is negligible. However, it makes a significant effect in the second half ($\tau_{40}$) of the nutrient release process as the degradation takes place by creating more pores in the coating, which encourages the fast release of nutrients from the granule. Hence, it has helped to understand the effect of reaction along with diffusion in the nutrient release profiles instead of dependence only on the diffusion coefficient and diffusion phenomenon as considered by Lu et al. [44].

### 3.2. Autocatalytic Concentration Profiles

The analytical expression gives the importance of first order reaction rates. This helps to understand whether the PLA degradation and erosion is increasing or decreasing by diffusion. However, the degradation reaction cannot fully complete as the ester bonds in the PLA are converted into the monomers after the hydrolysis reaction. The maximum time required for the hydrolysis reaction to take place can be calculated using Equation (24) [27]. 

The maximum time required to convert all the ester bond in the PLA to monomers is given below:

(24) $$t_{max}=\frac{1}{k}\ln\frac{M_{n0}}{M_{1}}$$

where $M_{n0}$ is the initial molecular weight and *M*_1_ is the average weight of monomers. Piemonte et al. [20] found that the hydrolytic degradation of PLA (*k* = 0.081 day^−1^) can be calculated at temperatures higher than 140 °C. Since the simulation carried out in the current study considers the PLA degradation in the aqueous environment, which is equal to the room temperature, so, we chose 0.015 day^−1^ as the initial point of the range of degradation. The autocatalytic concentration for degradation reaction without diffusion is described by [45]:

(25) $$\frac{\partial \theta_{r}}{\partial t}=k\theta_{r}$$

**Figure 5:**
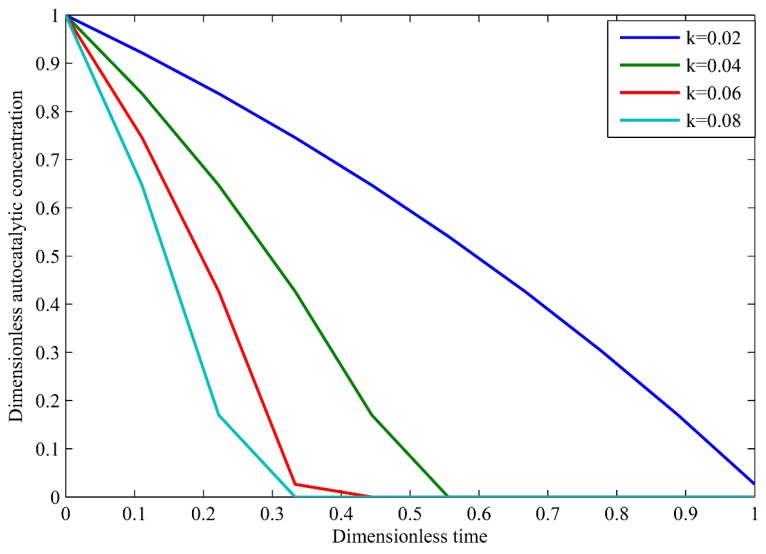
Dimensionless autocatalytic concentration versus dimensionless time for reactions.

The solution of Equation (25) is given below:

(25) $$\theta_{r}\left(r,t\right)=\theta_{r0}exp\left(kt\right)$$

where $\theta_{r}$ is referred as reaction dominant limit commonly used in autocatalytic degradation of PLA [46]. $\theta_{r0}$ is the initial concentration of monomers in the PLA coating undergoing hydrolytic degradation. The effect of reaction dominant limit and dimensionless concentration profile is shown in Figure 5.

**Figure 6:**
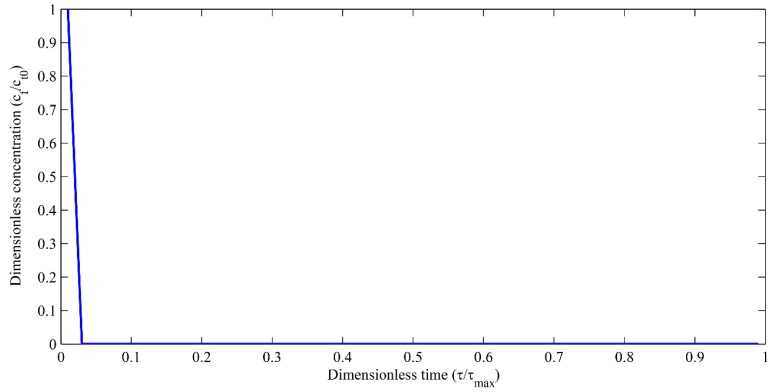
Dimensionless autocatalytic concentration versus dimensionless time for *k* = 0.05.

The dimensionless autocatalytic profiles of the reaction multi-diffusion equation has been obtained with time when$\;D_{r}=0.1$, $V_{r}=3$ and the space radial distance, η=2 and *k* = 0.05 day^−1^. The initial amount of monomers in the PLA, *C_t_*_0_ = 1.73 × 10^4^ mol/m^3^ [19]. As the degradation and diffusion dominate in the initial time until the monomers are converted into oligomers, the dimensionless autocatalytic concentration reduces exponentially. The concentration profiles have been taken at η=2 because after that the concentration gets diffused into the extraction medium. The simulation of dimensionless autocatalytic concentration profiles with dimensionless time are shown in Figure 6. 

**Figure 7:**
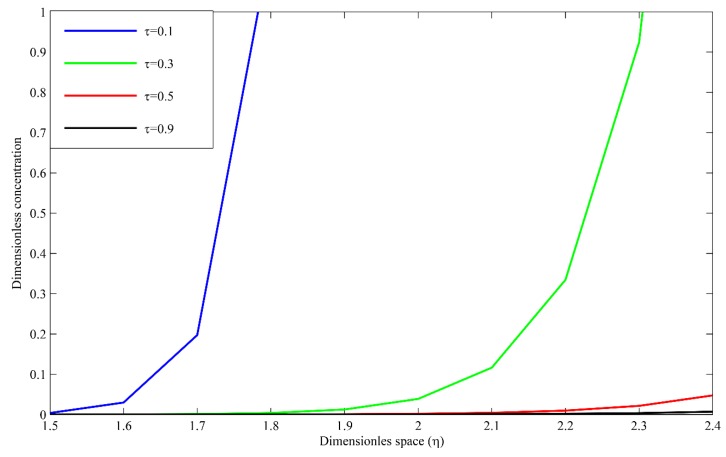
Dimensionless autocatalytic concentration profiles in the coating.

In Figure 7, the dimensionless autocatalytic concentration profiles are presented in the coating film with the distance at different dimensionless time. When *τ* = 0.1, the dimensionless concentration is at a maximum inside the coating. As the time increases, the concentration in the coating starts to reduce due to the reaction and diffusion phenomenon until η=2 when *τ* = 0.3. After that, there is an exponential reduction in the concentration as the oligomers are converted to monomers and concentration has dissolved in the extraction medium. This phenomenon can be inferred from the trend at *τ* = 0.5 and 0.9. 

The results for nutrient release profiles and autocatalytic concentration profiles suggest that it is important to consider the reaction-multi diffusion model to study the nutrient release characteristics. The autocatalytic degradation reaction has a significant effect on the release and concentration profiles. This provides further insight to design and develop biodegradable polymer-coated controlled-release fertilizers.

## 4. Conclusions

An analytical-numerical solution is presented to the reaction-multi diffusion model for a spherically-shaped PLA-coated CRF granule. We employed the Zakian method for the first time (to the best of the authors’ knowledge) for numerical inversion of the Laplace transformation that arises from the reaction-multi diffusion system. The expression gives an intuition of (i) nutrient release profiles and (ii) autocatalytic concentration profiles for different conditions, such as diffusion-only, both reaction and diffusion, and degradation-only reactions for the case of autocatalytic concentration. The limitation of this model occurs when the diffusion coefficient is considered as independent of the autocatalytic concentration profiles and time. Other degradation effects, such as enzymatic and microbial degradation have not been carried out but, in fact, it strongly affects the nutrient release.

The solution indicates that the reaction rate is a key parameter for the prediction of transition between diffusion and degradation of CRF system when autocatalytic concentration and nutrient release profiles are taken into account. The coupling effect of the reaction and diffusion system for nutrient release from PLA-coated controlled-release fertilizer is incorporated in this model. This helps to design and develop biodegradable polymer-coated CRF systems.

## Figures and Tables

**Table 1:** The values of $K_{j}$ and $\alpha_{j}$ for the Zakian method [40,42].

*j*	*K_j_*	*α_j_*
1	12.83767675 + 1.666063445*i*	−36,902.0821 + 196,990.4257*i*
2	12.22613209 + 5.012718792*i*	61,277.02524 – 95,408.62551*i*
3	10.93430308 + 8.409673116*i*	−28,916.56288 + 18,169.18531*i*
4	8.776434715 + 11.92185389*i*	4655.361138 − 1.901528642*i*
5	5.2254453361 + 15.72952905*i*	−118.7414011 − 141.3036911*i*

## Appendix A

By substituting Equations (14b) and (14c) into Equations (16) and (17) followed by simplifying, we get:

(A1a) $$\frac{1}{s}+A_{1}\sin h\sqrt{s}=K_{a}\left[A_{2}\sin h\sqrt{\frac{-k+s^{2}}{sD_{r}}}+B_{2}\cos h\sqrt{\frac{-k+s^{2}}{sD_{r}}}\right],$$

(A1b) $$A_{1}(\sqrt{s}\cos h\sqrt{s}-\sin h\sqrt{s})=D_{r}[A_{2}\left(\sqrt{\frac{-k+s^{2}}{sD_{r}}}\cos h\sqrt{\frac{-k+s^{2}}{sD_{r}}}-\sin h\sqrt{\frac{-k+s^{2}}{sD_{r}}}\right)+B_{2}\left(\sqrt{\frac{-k+s^{2}}{sD_{r}}}\sin h\sqrt{\frac{-k+s^{2}}{sD_{r}}}-\cos h\sqrt{\frac{-k+s^{2}}{sD_{r}}}\right)$$

Now, eliminating the $A_{1}\;$from Equations (A1a) and (A1b) results in:

(A2) $$A_{2}\left(K_{a}\sin h\sqrt{\frac{-k+s^{2}}{sD_{r}}}\left(\sqrt{s}\cos h\sqrt{s}-\sin h\sqrt{s}\right)-D_{r}\sin h\sqrt{s}(\sqrt{\frac{-k+s^{2}}{sD_{r}}}\cos h\sqrt{\frac{-k+s^{2}}{sD_{r}}}-\sin h\sqrt{\frac{-k+s^{2}}{sD_{r}}}\right)+B_{2}\left(K_{a}\cos h\sqrt{\frac{-k+s^{2}}{sD_{r}}}\left(\sqrt{s}\cos h\sqrt{s}-\sin h\sqrt{s}\right)-D_{r}\sin h\sqrt{s}(\sqrt{\frac{-k+s^{2}}{sD_{r}}}\sin h\sqrt{\frac{-k+s^{2}}{sD_{r}}}-\cos h\sqrt{\frac{-k+s^{2}}{sD_{r}}}\right)=\frac{\left(\sqrt{s}\cos h\sqrt{s}-\sin h\sqrt{s}\right)}{s}$$

From Equations (13d) and (13e), after eliminating ${\bar{\theta }}_{e}$ follow by simplifying using the Equation (15), we get:

(A3) $$A_{2}\left[\left(\sqrt{\frac{-k+s^{2}}{sD_{r}}}l\cos h\sqrt{\frac{-k+s^{2}}{sD_{r}}}l-\sin h\sqrt{\frac{-k+s^{2}}{sD_{r}}}l\right)+\frac{V_{r}K_{b}l^{2}}{3}\left(\frac{-k+s^{2}}{sD_{r}}\right)\sin h\sqrt{\frac{-k+s^{2}}{sD_{r}}}l\right]+B_{2}\left[\left(\sqrt{\frac{-k+s^{2}}{sD_{r}}}l\sin h\sqrt{\frac{-k+s^{2}}{sD_{r}}}l-\cos h\sqrt{\frac{-k+s^{2}}{sD_{r}}}l\right)+\frac{V_{r}K_{b}l^{2}}{3}\left(\frac{-k+s^{2}}{sD_{r}}\right)\cos h\sqrt{\frac{-k+s^{2}}{sD_{r}}}l\right]=0$$

Equations (A2) and (A3) can be further simplified and written in the form of the matrix as follows:

(A4) $$\left[\begin{array}{cc} g_{1}\left(s\right) & g_{2}\left(s\right)\\ g_{3}\left(s\right) & g_{4}\left(s\right) \end{array}\right]\left[\begin{array}{c} A_{2}\\ B_{2} \end{array}\right]=\left[\begin{array}{c} \frac{\left(\sqrt{s}\cos h\sqrt{s}-\sin h\sqrt{s}\right)}{s}\\ 0 \end{array}\right]$$

where:

(A5a) $$g_{1}\left(s\right)=K_{a}\sin h\sqrt{\frac{-k+s^{2}}{sD_{r}}}\left(\sqrt{s}\cos h\sqrt{s}-\sin h\sqrt{s}\right)-D_{r}\sin h\sqrt{s}(\sqrt{\frac{-k+s^{2}}{sD_{r}}}\cos h\sqrt{\frac{-k+s^{2}}{sD_{r}}}-\sin h\sqrt{\frac{-k+s^{2}}{sD_{r}}}$$

(A5b) $$g_{2}\left(s\right)=K_{a}\cos h\sqrt{\frac{-k+s^{2}}{sD_{r}}}\left(\sqrt{s}\cos h\sqrt{s}-\sin h\sqrt{s}\right)-D_{r}\sin h\sqrt{s}(\sqrt{\frac{-k+s^{2}}{sD_{r}}}\sin h\sqrt{\frac{-k+s^{2}}{sD_{r}}}-\cos h\sqrt{\frac{-k+s^{2}}{sD_{r}}}$$

(A5c) $$g_{3}\left(s\right)=\left(\sqrt{\frac{-k+s^{2}}{sD_{r}}}l\cos h\sqrt{\frac{-k+s^{2}}{sD_{r}}}l-\sin h\sqrt{\frac{-k+s^{2}}{sD_{r}}}l\right)+\frac{V_{r}K_{b}l^{2}}{3}\left(\frac{-k+s^{2}}{sD_{r}}\right)\sin h\sqrt{\frac{-k+s^{2}}{sD_{r}}}l$$

(A5d) $$g_{4}\left(s\right)=\left(\sqrt{\frac{-k+s^{2}}{sD_{r}}}l\sin h\sqrt{\frac{-k+s^{2}}{sD_{r}}}l-\cos h\sqrt{\frac{-k+s^{2}}{sD_{r}}}l\right)+\frac{V_{r}K_{b}l^{2}}{3}\left(\frac{-k+s^{2}}{sD_{r}}\right)\cos h\sqrt{\frac{-k+s^{2}}{sD_{r}}}l$$

(A6a) $$A_{2}=\frac{\left(\sqrt{s}\cos h\sqrt{s}-\sin h\sqrt{s}\right)g_{4}\left(s\right)}{s\left(g_{1}\left(s\right)g_{4}\left(s\right)-g_{2}\left(s\right)g_{3}\left(s\right)\right)},$$

(A6b) $$B_{2}=\frac{\left(\sqrt{s}\cos h\sqrt{s}-\sin h\sqrt{s}\right)g_{3}\left(s\right)}{s\left(g_{1}\left(s\right)g_{4}\left(s\right)-g_{2}\left(s\right)g_{3}\left(s\right)\right)}$$

After substituting Equations (A6a) and (A6b) into Equation (A1b), the value of $A_{1}$ is given below:

(A7) $$A_{1}=\frac{D_{r}}{s\left(g_{1}\left(s\right)g_{4}\left(s\right)-g_{2}\left(s\right)g_{3}\left(s\right)\right)}[\left(l-1\right)\sqrt{\frac{-k+s^{2}}{sD_{r}}}+\frac{V_{r}K_{b}l^{2}}{3}\left(\frac{-k+s^{2}}{sD_{r}}\right)\sqrt{\frac{-k+s^{2}}{sD_{r}}}\cos h\sqrt{\frac{-k+s^{2}}{sD_{r}}}\left(l-1\right)+l\left(\frac{-k+s^{2}}{sD_{r}}\right)-1+\frac{V_{r}K_{b}l^{2}}{3}\left(\frac{-k+s^{2}}{sD_{r}}\right)\sin h\sqrt{\frac{-k+s^{2}}{sD_{r}}}\left(l-1\right)]$$
